# Supplementary material for: Identification of photoperiod-induced specific miRNAs in the adrenal glands of Sunite sheep (Ovis aries)
Source: Front Vet Sci. 2022 Jul 22;9:888207. doi: 10.3389/fvets.2022.888207 (PMC9354845; doi:10.3389/fvets.2022.888207)
Supplement: Supplementary file 1 [file Data_Sheet_1.ZIP › Supplementary file/miRDeep2 file.zip!/miRDeep2 file/Novel_Structure/SP42A1/Novel_326.pdf]

Provisional ID : NC_019471.2_17281
Score total : 1.9
Score for star read(s) : -1.3
Score for read counts : 0
Score for mfe : 1.6
Score for randfold : 1.6
Score for cons. seed :
Total read count : 28
Mature read count : 28
Loop read count : 0
Star read count : 0

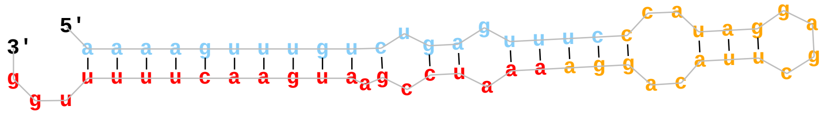

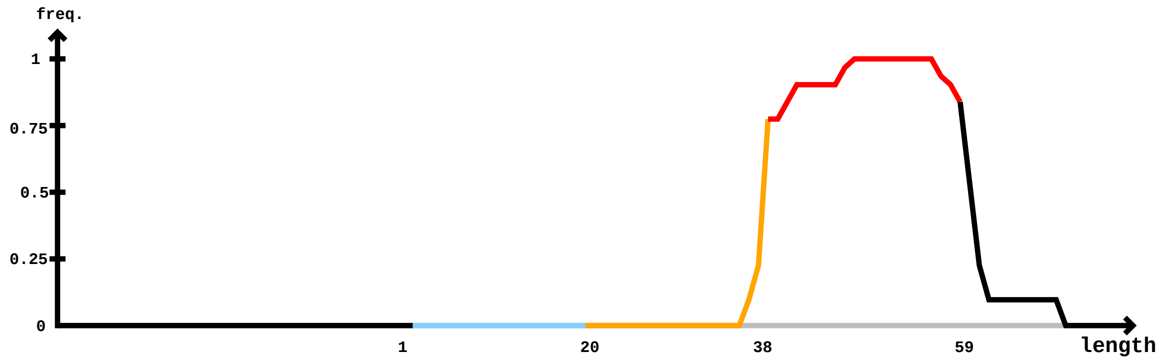

Star Mature

```
5'- cuuuuaaauuuaugcacauauguauuggguuggccaaaaguuugucugaguuucccauaggagcuuacaggaaaauccgaaugaacuuuuuggccaacccauauauuggc   -3'   exp
    .............((.((.((((((.((((((((((((((((((((.((.(((((..(((....)))..))))).)).).)))))).)))))))))))))))))))))))   reads   mm   sample
    ......................................................................gaaaauccgaaugaacuuuu....................   1    0    seq
    ......................................................................gaaaauccgaaugaacuuuuu...................   1    0    seq
    ......................................................................gaaaauccgaaugaacuuuuug..................   1    0    seq
    .......................................................................aaaauccgaaugaacuuuu....................   1    0    seq
    .......................................................................aaaauccgaaugaacuuuuug..................   1    0    seq
    .......................................................................aaaauccgaaugaacuuuuugg.................   2    0    seq
    ........................................................................aaaCccgaaugaacuuuuugg.................   17   1    seq
    ..........................................................................auccgaaugaacuuuuuggUc...............   1    1    seq
    ..........................................................................aCccgaaugaacuuuuuggcc...............   1    1    seq
    ...........................................................................uccgaaugaacuuuuuggUc...............   2    1    seq
    ................................................................................augaacuuuuuggccaacccaua.......   1    0    seq
    ................................................................................augaacuuuuuggccaacccaGa.......   1    1    seq
    .................................................................................ugaacuuuuugAccaacccaua.......   1    1    seq
```
